# AI lesion tracking in PET/CT imaging: a proposal for a Siamese-based CNN pipeline applied to PSMA PET/CT scans

**DOI:** 10.1007/s00259-025-07426-5

**Published:** 2025-07-08

**Authors:** Stefan P. Hein, Manuel Schultheiss, Andrei Gafita, Raphael Zaum, Farid Yagubbayli, Robert Tauber, Isabel Rauscher, Matthias Eiber, Franz Pfeiffer, Wolfgang A. Weber

**Affiliations:** 1 Department of Nuclear Medicine, School of Medicine and Health, TUM Klinikum, Technical University of Munich, Munich, 81675 Germany; 2 Chair of Biomedical Physics, Department of Physics, TUM School of Natural Sciences, Technical University of Munich, Garching, 85748 Germany; 3 Institute for Diagnostic and Interventional Radiology, School of Medicine and Health, TUM Klinikum, Technical University of Munich, Munich, 81675 Germany; 4 Division of Nuclear Medicine and Molecular Imaging, The Russell H. Morgan Department of Radiology and Radiological Science, Johns Hopkins University School of Medicine, Baltimore, 21205 MD USA; 5 Department of Urology, School of Medicine and Health, TUM Klinikum, Technical University of Munich, Munich, 81675 Germany; 6 Munich Institute of Biomedical Engineering, Technical University of Munich, Garching, 85748 Germany

**Keywords:** PET/CT, Lesion tracking, AI, CNN, PSMA, Metastatic castration resistant prostate cancer

## Abstract

**Purpose:**

Assessing tumor response to systemic therapies is one of the main applications of PET/CT. Routinely, only a small subset of index lesions out of multiple lesions is analyzed. However, this operator dependent selection may bias the results due to possible significant inter-metastatic heterogeneity of response to therapy. Automated, AI-based approaches for lesion tracking hold promise in enabling the analysis of many more lesions and thus providing a better assessment of tumor response. This work introduces a Siamese CNN approach for lesion tracking between PET/CT scans.

**Methods:**

Our approach is applied on the laborious task of tracking a high number of bone lesions in full-body baseline and follow-up [^68^Ga]Ga- or [^18^F]F-PSMA PET/CT scans after two cycles of [^177^Lu]Lu-PSMA therapy of metastatic castration resistant prostate cancer patients. Data preparation includes lesion segmentation and affine registration. Our algorithm extracts suitable lesion patches and forwards them into a Siamese CNN trained to classify the lesion patch pairs as corresponding or non-corresponding lesions.

**Results:**

Experiments have been performed with different input patch types and a Siamese network in 2D and 3D. The CNN model successfully learned to classify lesion assignments, reaching an accuracy of 83 % in its best configuration with an AUC = 0.91. For corresponding lesions the pipeline accomplished lesion tracking accuracy of even 89 %.

**Conclusion:**

We proved that a CNN may facilitate the tracking of multiple lesions in PSMA PET/CT scans. Future clinical studies are necessary if this improves the prediction of the outcome of therapies.

**Supplementary Information:**

The online version contains supplementary material available at 10.1007/s00259-025-07426-5.

## Introduction

 The high sensitivity and specificity of PSMA-PET/CT scans in detecting lesions [1] offers an enormous opportunity for accurate response monitoring and the detection of disease progression or regression. However, this creates new challenges to be solved. Especially prostate cancer patients frequently show diffuse bone lesion patterns with a high number of tracer uptake foci to be examined [2]. Since detailed manual lesion tracking is laborious and error-prone, routinely, only a small subset of index lesions out of multiple lesions is analyzed [3]. However, the operator dependent selection of index lesions may bias the results due to possible significant inter-metastatic heterogeneity of response to therapy. Imaging with PSMA-radioligands might require a deeper and broader lesion analysis.

Also, a mere extraction of overall quantitative features like the PSMA-positive total tumor volume might give a summary of the general prostate cancer development but does not account for varying cancer evolution patterns in the different organs, which can be especially important after a combination of systemic and local therapy. Also, it has been shown that the PSMA-positive total tumor volume is not sufficient to predict progression or response to therapy without taking into account new lesions [4]. Lesion tracking may enable both the automatic detection of new lesions as well as analyzing the heterogeneous lesion development in different body regions. In a clinical workflow, a lesion tracking algorithm bears the chance of a more exact assessment of the response to therapy and might provide automation support for quickly comparing the scans. Thus, reporting on a detailed level could be handled more efficiently.

Studies have shown that multivariable models considering tumor lesion heterogenity in response evaluation of prostate cancer patients lead to better results in assessing patient outcomes than univariate analyses [5]. New automated and AI-based approaches for lesion tracking might enable the analysis of even more lesions providing a better assessment of tumor response and prediction of patient outcome. Given the high number of PSMA-uptake foci, automated tracking in PET/CT scans is particularly useful for prostate cancer bone lesions. Therefore, our focus is on this application for our new tracking approach.

So far, only few approaches exist for automated lesion tracking specifically in PET/CT scans, mostly relying on image registration. Opfer et al. were among the first to suggest a tracking algorithm for lesions in PET/CT scans based on a global rigid registration, block matching and region growing [6]. Fox et al. also proposes comparative analysis approach including a registration-based propagation of a selected region into the follow-up PET/CT scan [7]. For registration-based bone lesion matching, a human skeleton articulated registration algorithm has been proven to yield good results [8]. More recent developments have been presented by Santoro-Fernandes et al. with automated lesion tracking for lesions in FDG (fluorodeoxyglucose) and FLT (fluorothymidine) PET/CT based on image registration, lesion dilation and a lesion clustering concept assigning the lesions [9]. The approach has been later extended to lesions in PET/CT and PET/MR for several imaging time points in neuroendocrine tumor patients [10]. In those works, lesions have been segmented manually [9, 10].

To our best knowledge, there is yet no tracking algorithm for PET/CT applying artificial intelligence, unlike for segmentation or localization of PET lesions [11–13].

In contrast to PET/CT scans, lesion tracking has been more studied for other imaging modalities, as ultrasound or stand-alone CT, also often based only on registration [14]. Within ultrasound, MRI or CT images, however, CNNs (Convolutional Neural Networks) have already been applied. In Dankerl et al. [15] a pattern recognition network detects landmarks and localizes follow-up lesions based on a patient specific graphical network. Furthermore, Hering et al. use a nnU-Net for baseline CT lesion segmentation and perform lesion tracking by segmenting lesions in the registration-based propagated ROI of the follow-up CT scan [16]. Kuckertz et al. also use a U-Net based lesion segmentation and non-liniear image registration for tracking of FLAIR MRI multiple sclerosis lesions [17] and CT liver lesions [18].

In case deep learning is directly applied for lesion tracking, it is mostly in the shape of Siamese networks. In ultrasound images, they are applied by Gomariz et al. [19] and Liu et al. [20] for liver landmark and liver respiratory motion tracking. In CT scans, Rafael-Palou et al. [21] uses a 3D Siamese network for lung nodule tracking and Cai et al. [22] extend a 3D Siamese network in depth for CT lesion tracking.

Other network types have been explored for lesion tracking in CT by Tang et al. with a Transformer network [23] and by Szeskin et al. with a SimU-Net [24]. Rochman et al. extended the SimU-Net approach to handle more complex lesion changes [25].

In this work, we aim to fill the gap of a general AI lesion tracker for PET/CT scans, by proposing a whole pipeline of automated registration, lesion segmentation and tracking based on a Siamese CNN structure in two and three dimensions. It is bootstrapped on the use case of numerous bone lesions in PSMA PET/CT images of metastatic castration resistant prostate cancer patients. The objective of the study was to provide proof-of-concept data for tracking a large number of lesions on PSMA PET scans over time using a Siamese CNN. The performance of the AI algorithm was evaluated based on its ability to identify corresponding lesions on two scans, as well as to detect new and disappeared lesions - including more complex scenarios such as lesion coalescence or fragmentation.

## Methods

### Workflow overview

**Fig. 1:**
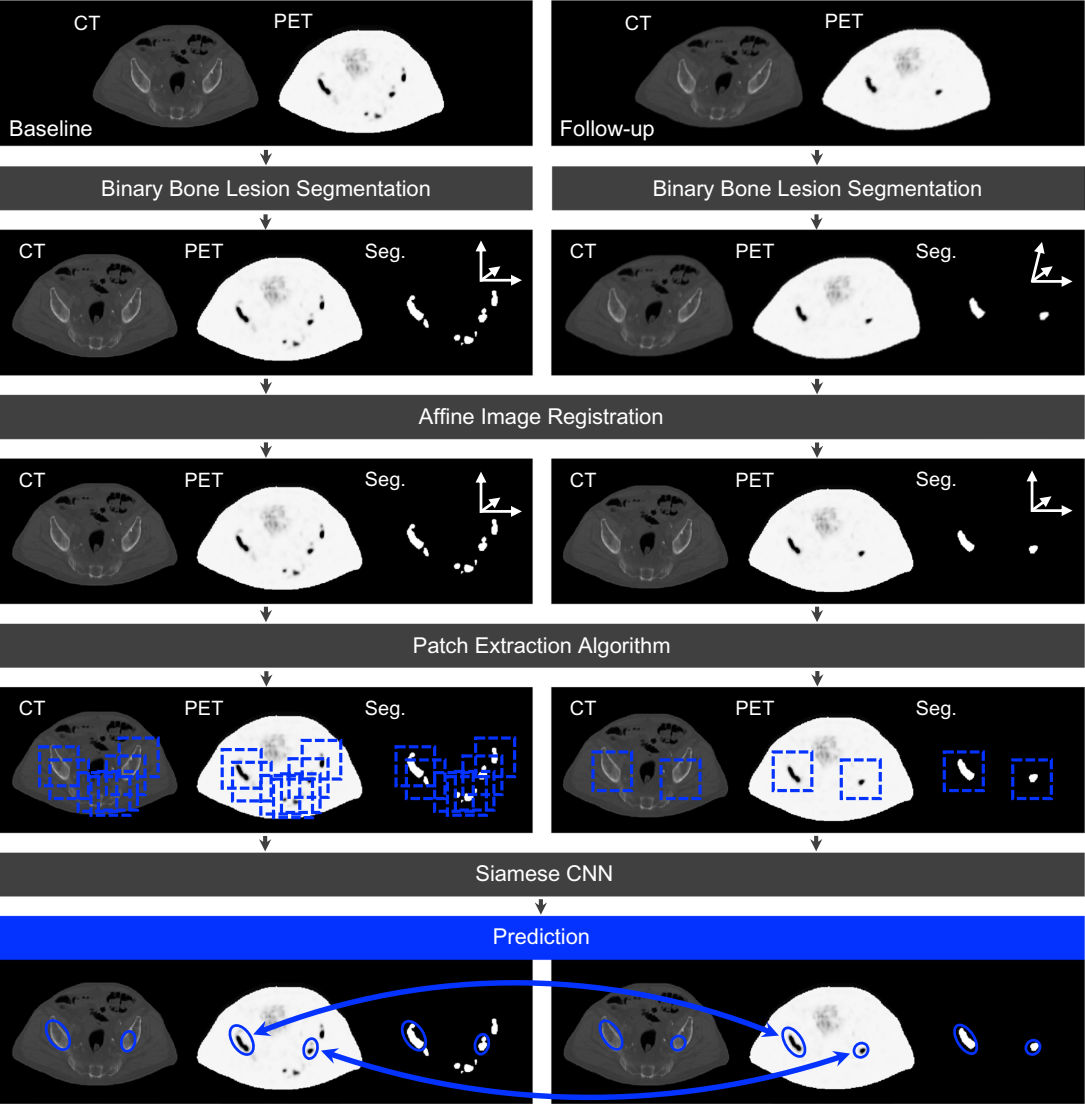
Processing workflow. Schematic presentation of the proposed pipeline for AI-based PET/CT lesion tracking. Data preparation of the baseline and follow-up scans include a binary bone lesion segmentation (Seg.) and an affine image registration. Patches are extracted around every lesion and then analyzed by a siamese CNN, which assigns the lesions in the baseline and follow-up PET/CT scans

The workflow is displayed in Fig. 1. Experiments have been performed in two and three dimensions with 2D and 3D network architectures and patches. Each baseline and follow-up full-body CT and PET scan in DICOM format was processed by the qPSMA software [26], to create a lesion segmentation mask. This algorithm extracts bone lesions by applying an SUV threshold within a segmented bone mask, taking into account a possible misalignment of PET and CT data.

As the patient’s position can be shifted in follow-up scans, a rough alignment is required for relocalization during later patch extraction. Therefore an affine registration is applied (Supplements: Image Registration). For image registration SimpleElastix [27] was used.

**Fig. 2:**
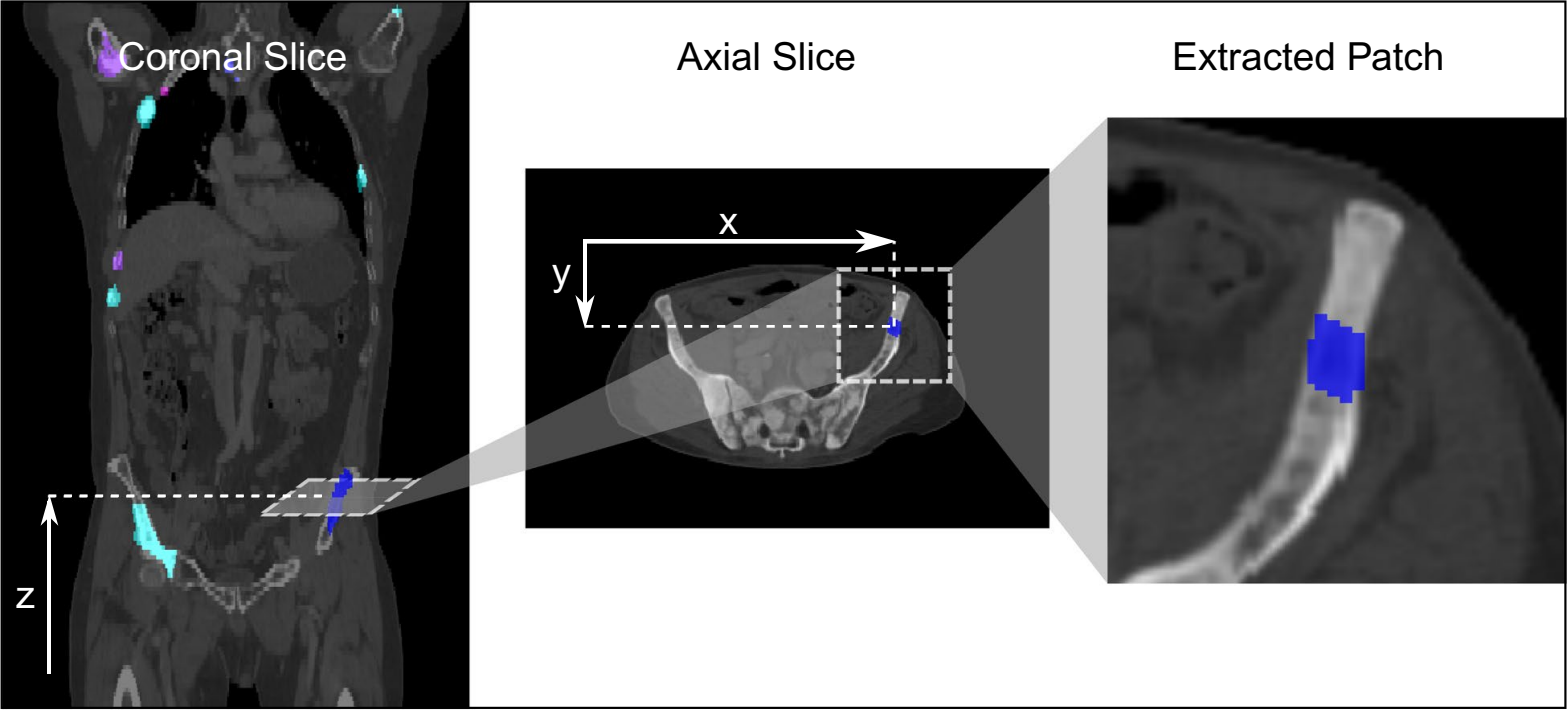
2D lesion patch extraction. An axial 50$\times$50 pixel patch is cropped around the determined extraction point *p*(*x*, *y*, *z*)

**Fig. 3:**
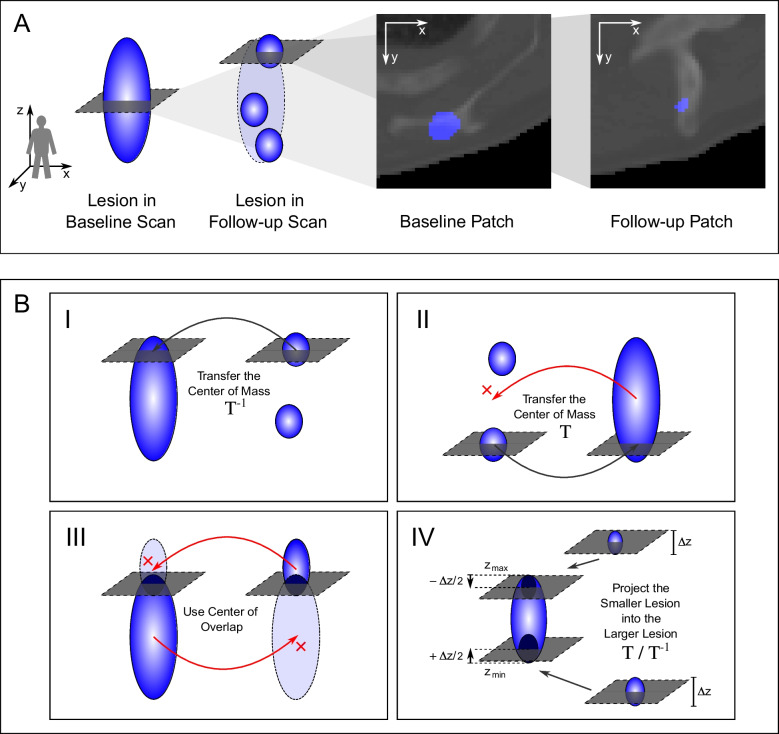
Principle of the patch extraction algorithm. **A** Unsuitable patch pair after extraction at center of mass. A large baseline lesion shrank and divided into three smaller lesions in the follow up-scan. The example shows a baseline and follow-up lesion patch of the left scapula extracted at the lesions’ center of mass (CoM). Patch extraction at each lesion’s CoM can lead to patch pairs showing different anatomical environments, even though they show corresponding lesions. For this reason, a detailed patch extraction algorithm determines the suitable patch extraction point *p*(*x*, *y*, *z*). **B** Patch extraction cases. In the algorithm a case distinction hierarchically applies the four cases I-IV to find suitable points *p*(*x*, *y*, *z*) within the baseline and follow-up lesion for the patch pair extraction. If one case does not lead to a result, the algorithm passes on to the next one. For each case, the illustration shows the baseline lesion(s) on the left side and the follow-up lesion(s) on the right side. The gray cross-sectional areas indicate the final extracted axial patches. $\textbf{T}$ and $\mathbf {T^{-1}}$ indicate point transfers to the respective other scan (Supplements: Eqs. 1, 2). The cases are described in detail in the supplements

In a next step image patches are cropped around every lesion and its surrounding region in the baseline and the follow-up scan (Fig. 2). Patches are extracted in 2D with a size of 50x50 pixels and in 3D with 50x50x5 and 50x50x11 pixels. A sufficient patch size had been estimated by visually analyzing the axial lesion dimensions. The suitable extraction positions are determined by a detailed case distinction algorithm, which already processes the three-dimensional relation, as cropping the patches directly from the Center of Mass (CoM) of each lesion can lead to anatomically non-related patches (Fig. 3, for details see Supplements: Patch Extraction).

The 2D or 3D Siamese CNN (Section Siamese CNN) compares pairs of baseline and follow-up lesion patches and decides, whether the patch pair shows corresponding lesions. The approach narrows the tracking down to a classification problem: true patch pair for corresponding lesions, false patch pair for non-corresponding lesions.

### Siamese CNN

**Fig. 4:**
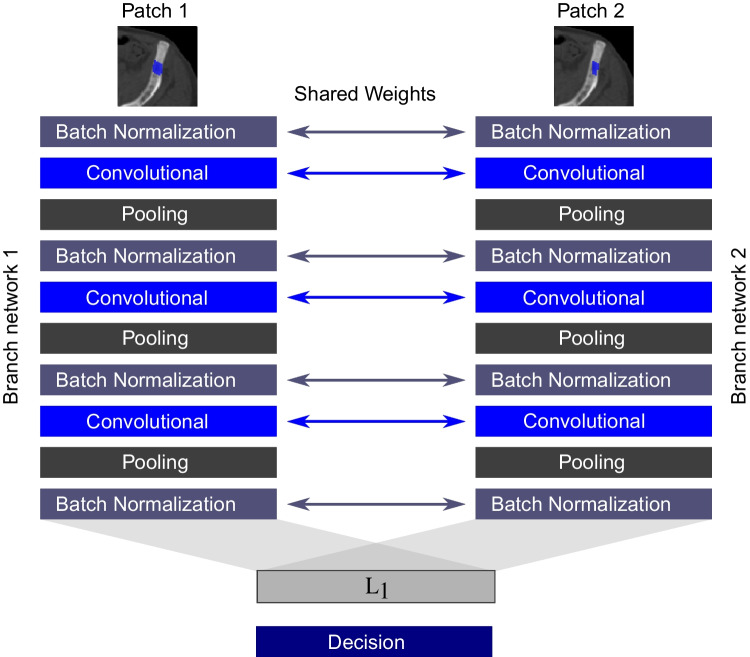
Structure of the siamese network. Patch 1 displays a baseline lesion and Patch 2 shows a follow-up lesion. The patches are processed by two parallel CNN branches with shared weights, whose output is merged by a $\mathbf {L_1}$-layer

A Siamese network consisting of two parallel CNN branches was chosen to process the input of patch pairs (Fig. 4). It uses shared weights for the two branches and was inspired by Koch et al. [28]. Each CNN branch has three convolutional layers with *ReLU* activation and three pooling layers. Batch normalization is applied before every convolutional layer. The output of a branch again undergoes batch normalization, is flattened to a vector $\textbf{h}(\textbf{X})$ and is merged in a custom-defined $\mathbf {L_1}$-layer, which takes the element-wise absolute difference of the feature vectors $\textbf{h}(\mathbf {X_1})$ and $\textbf{h}(\mathbf {X_2})$:

1 $$\begin{aligned} \mathbf {L_1}(\mathbf {X_1}, \mathbf {X_2})_{i}=|| \textbf{h}(\mathbf {X_1})_i - \textbf{h}(\mathbf {X_2})_i||_1 \text {.} \end{aligned}$$

The output $\mathbf {L_1}(\mathbf {X_1}, \mathbf {X_2})$ is fed to a final fully-connected decision layer (dense layer) and a *softmax function* distributing the final network output to the two classes *true patch pair* and *wrong patch pair*. Details of the branch architecture are displayed in supplemental Table 1.

To process the different patch dimensions (2D, 3D), the Siamese network was created in 2D and 3D. Additionally, the *Adam* optimizer [29], *learning rate scheduling* [28, 30, 31], $\ell ^2$*-parameter regularization* [32] and a *dropout rate* [33] have been applied as recommended for siamese networks (for details see Supplements: Siamese Network).

### Experimental setup

Lesions may evolve along different patterns, which have to be recognized by the pipeline. A lesion can be persisting in both scans as a single lesion, split into several lesions or fuse with neighboring lesions. Also, a baseline lesion can resolve completely or a new lesion can develop in the follow-up.

The Siamese CNN is trained with balanced ground truth data containing equal numbers of true patch pairs and false patch pairs. For the application of the network on a test set, all possibly relevant lesion combinations are fed to the CNN in the shape of patch pairs. The predictions are used to assign the lesions. If all patch pairs of a baseline lesion are classified as false patch pairs, this lesion can be declared as disappeared and no follow-up lesions are assigned. The same situation for a lesion from the follow-up scan suggests that it is a new lesion.

Since whole-body imaging data is available from CT, PET and binary lesion segmentation, all of it can serve as an input for the patch extraction. Therefore, we processed four different patch types in the network, single-channel CT patches and several two-channel patches that combined CT & PET, CT & Binary Lesion Segmentation and CT & Segmented CT data. As *Segmented CT* we define the element-wise product of the Binary Lesion Segmentation patch with the CT patch, showing the CT information only within the segmented lesion.

Experiments have been performed with 2D and 3D networks and corresponding patches.

### Dataset

In this project, the processed dataset consists of 36 patients with metastatic castration resistant prostate cancer with a high number of bone lesions requiring follow-up tracking. The patients were treated with [^177^Lu]Lu-PSMA-I&T (imaging and therapy) [34] and a total number of 188 cycles (median 5 cycles, range 1–31). Eligibility criteria were previous treatment with abiraterone or enzalutamide, previous taxane-based chemotherapy or chemoineligibility, and positive PSMA-ligand uptake at PET scan. The [^177^Lu]Lu-PSMA-I&T was given 6–8 weekly with an activity of 7.4 GBq in up to six cycles in a row with a potential continuation after a therapy break. Within the selected patient group, prostate-specific antigen decline of $>50\%$ was achieved in 15 patients (43 %), median PSA-progression-free survival was 5.3 months, and median overall survival (OS) was 14.2 months.

For each patient a full-body baseline and a follow-up [^68^Ga]Ga-PSMA-11 [35] or [^18^F]F-rhPSMA-7 [36, 37] PET/CT scan after two cycles of [^177^Lu]Lu-PSMA-I&T [34] therapy are analyzed, adding up to 2111 baseline lesions and 2658 separately segmented foci in the follow-up scans. The baseline and follow-up lesions show 1490 true lesion assignments, in average 41.4 pairs of corresponding lesions per patient. The average number of lesions to analyze per scan was 57.6.

The PET/CT tracers were administered in compliance with the German Medicinal Products Act, AMG x13(2b), and in accordance with the responsible regulatory body (Government of Oberbayern). They were synthesized and all scans performed at our institution as described in Eiber et al. [38] and Kroenke et al. [39]. For the image acquisition the scanners Siemens Biograph mCT and Siemens Biograph Vision were used. After image reconstruction, the transaxial pixel size was 4.07 mm for PET and 1.52 mm for CT, each with a 5 mm or 3 mm slice thickness.

Ground truth lesion pairs were manually assigned by an experienced nuclear medicine physician using an in-house software. The dataset was split on a patient level into 70% training set, 15% validation set and 15% test set. The distribution was applied in a way that ensured this ratio for the patients as well as for the lesions. In addition, data augmentation in form of patch rotation was implemented to further increase the size of the training dataset by a factor of 10.

### Performance analysis

All runs of the training process have been conducted several times on the training set with different seeds to show statistical stability. In parallel to the training a first testing has been performed on the validation set. Every training has been carried out for 400 epochs. We chose the lowest validation loss as criterion to select the epoch with the best weights of the model.

In a final step the trained model has been applied on the separated test set. During testing, as default, a threshold of 0.5 is applied for the classification problem with the two classes true patch pair and false patch pair.

The ROC (Receiver operating characteristic) curve and the corresponding AUC (Area under the curve) has been used for rating the CNN performance. The statistical significance of the differences in the results has been analyzed by pairwise comparison of the AUC values according to the De Long’s test. A $p < 0.05$ is considered significant.

After selecting the best performing model, to obtain the best achievable result indicated by the upper left of the ROC, we implemented threshold optimization by maximizing $G_{\text {mean}}$, defined as

2 $$\begin{aligned} G_{mean} = \sqrt{sens(thr) \times spec(thr) \ } \end{aligned}$$

with *sens*(*thr*) and *spec*(*thr*) as the sensitivity and specificity in dependence of the selected threshold *thr*.

## Results

For each combination of 2D or 3D Siamese CNN and the different patch types, training and testing has been performed. The detailed performance result on the validation set during training and on the test set is displayed in supplemental Table 2.

On the test set, the 2D Siamese CNN outperforms for most of the patch types. Within the same network type, training with the single-channel CT patches reaches the best test accuracy, especially for the 2D network (83 %) and the 3D Siamese CNN with 50$\times$50$\times$5 patches (82 %). Considering the standard deviation, the CT patches also work amongst the best in the 3D network with 50$\times$50$\times$11 patches.

**Fig. 5:**
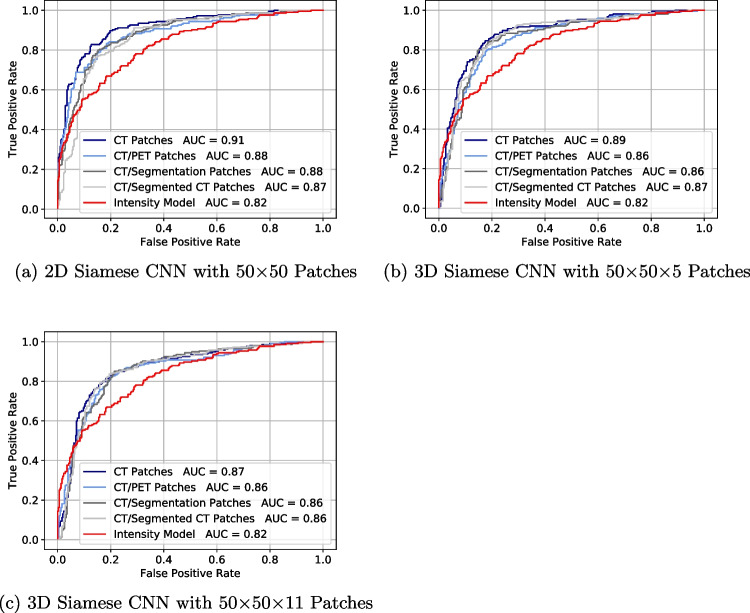
ROC curves of the siamese networks trained with different patch types. The AUC values for each curve are given in the legend. The best network configuration is reached with a 2D Siamese network trained with single-channel CT patches (AUC=0.91). The non-trainable reference intensity model (Supplements: Reference Model) reaches an AUC=0.82, which is outperformed by all networks and patch types

The ROC curves of the differently trained networks are shown in Fig. 5 and confirm the described results. As a reference model, all graphs show the ROC of a non-trainable intensities model (Supplements: Reference Model). All trained networks outperform the intensity model significantly proving a successful training process. Clearly, the 2D Siamese network trained with single-channel CT patches reaches the best result with an AUC = 0.91, which is significantly higher than for all other combinations of networks and patch types ($p < 0.05$). The resulting *p*-values of the statistical comparison of the best AUC values according to the pairwise De Long’s test are given in Table 1.

**Table 1:** Statistical comparison of the network-patch type combinations with the highest SUV according to the De Long’s test, with the highest AUC (2D CNN with CT patches) as a reference. All comparisons are significant with $p < 0.05$

Comparison of network-patch type combinations	AUC	*p*-value
2D CNN with CT patches - 2D CNN with CT/PET patches	$0.91 - 0.88$	0.008
2D CNN with CT patches - 2D CNN with CT/Segmentation patches	$0.91 - 0.88$	0.007
2D CNN with CT patches - 2D CNN with CT/Segmentation patches	$0.91 - 0.88$	0.001
2D CNN with CT patches - 2D CNN with CT/Segmented CT patches	$0.91 - 0.87$	$<0.001$
2D CNN with CT patches - 3D CNN with CT 50x50x5 patches	$0.91 - 0.89$	0.028
2D CNN with CT patches - 3D CNN with CT 50x50x11 patches	$0.91 - 0.87$	0.001

**Fig. 6:**
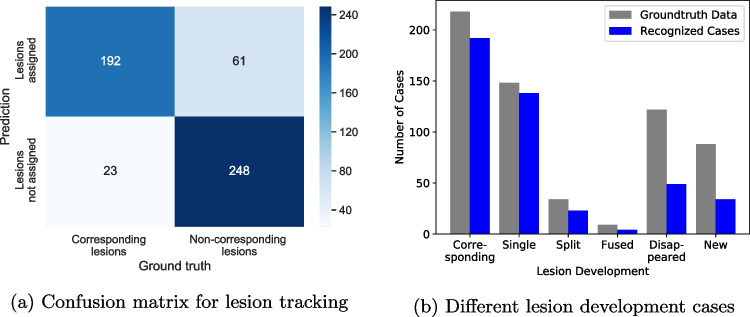
Pipeline Performance. **a** The figure illustrates the number of true positives (upper left), false positives (upper right), false negatives (lower left) and true negatives (lower right) among a cumulative total of 524 decisions by the 2D siamese network trained with 50$\times$50 CT patches. Threshold optimization was performed by $G_{\text {mean}}$ maximization. **b** Pipeline performance for the corresponding lesion cases, with the subcategories of corresponding lesions persisting as single lesions, split lesion cases and fused lesion cases, and for the cases of disappeared lesions and newly developed lesions

Table 2 displays the statistical parameters before and after threshold optimization by $G_{\text {mean}}$ maximization. The final model reaches an 83% accuracy, a 88% sensitivity, a 80% specificity and a 76% precision. This results in a $G_{\text {mean}}$ of 0.84 and a $F_1$-score of 0.82. The resulting confusion matrix with a total number of 524 decision cases in the test set is shown in Fig. 6a.

**Table 2:** Statistical parameters for the performance of the 2D Siamese network trained with 50$\times$50 CT patches

Threshold	Accuracy	Sensitivity	Specificity	Precision
Default 0.5	$0.830 \pm 0.003$	$0.731 \pm 0.039$	$0.910 \pm 0.032$	$0.850 \pm 0.037$
$G_{\text {mean}}$ optimized	$0.833 \pm 0.006$	$0.881 \pm 0.021$	$0.800 \pm 0.016$	$0.755 \pm 0.012$

As a last step of the pipeline, the results of the network decisions are used to determine if baseline lesions persist, resolve or newly appear in the follow-up. The pipeline performance on the detection of the different lesion development cases are shown in Fig. 6b. Corresponding (persisting) lesions are tracked successfully in 89.3% of the cases. Amongst them, baseline lesions persisting as single lesions in the follow-up are recognized with a 93.2% success rate, whereas splitting cases are recognized in 67.6% and fused lesions in 44.4% of the cases. Disappeared baseline lesions are correctly identified in 40.2% and new follow-up lesions in 38.6% of the instances.

**Fig. 7:**
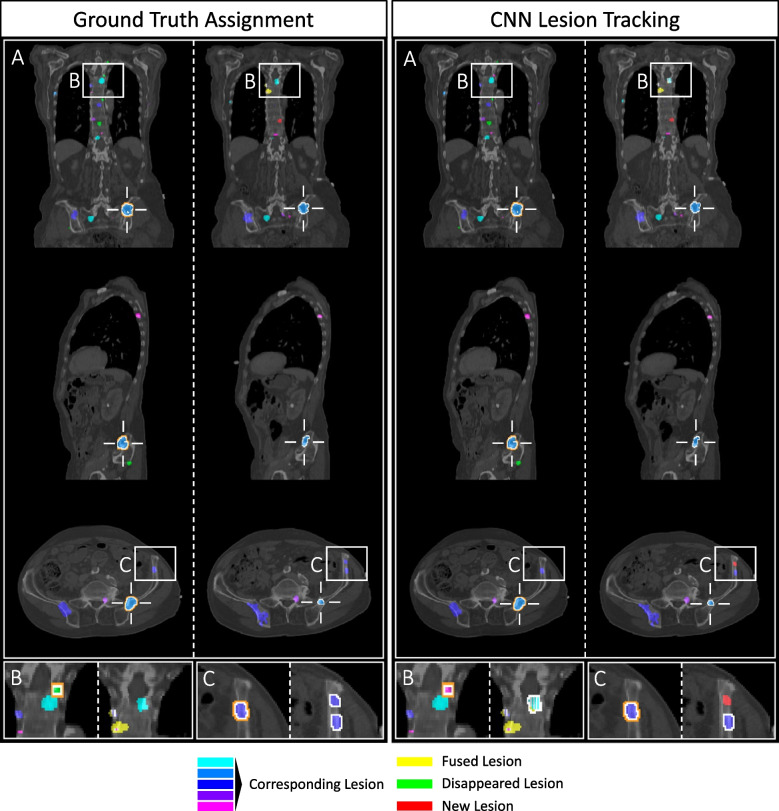
Application of CNN lesion tracking on PSMA-PET/CT scans of a prostate cancer patient. Comparison of the nuclear medicine specialist’s ground truth lesion assignment (left column) to the result of CNN lesion tracking (right column), with each the baseline scan on the left side of the dotted line and the follow-up on the right side. Baseline and follow-up lesions with the same color have been assigned as corresponding lesions, fused lesions are marked yellow, disappeared lesions green and new lesions red. **A** 3D overview of the lesion distribution and the tracking result: Exemplary, the coronal, sagittal and axial view display planes of the lesion indicated with the white cross. All shown lesions except one (B) have been correctly tracked by the CNN. **B** Disappeared lesion closely located to a persisting lesion: The CNN has wrongly classified the disappeared lesion as a fusion with the persisting lesion due to its close anatomic spatial relation. Often, these cases even show a high inter-observer variability amongst nuclear medicine specialists. **C** CNN outperforms nuclear medicine specialist: The ground truth set shows a wrongly annotated split lesion. However, the CNN suggested the second follow-up lesion to be a new lesion. After reviewing the PET dataset, the CNN’s decision has been proven correct

An application example of Siamese CNN lesion tracking in two consecutive scans of a patient is shown in Fig. 7, where the CNN in one lesion tracking case even outperforms the nuclear medicine specialist (Fig. 7C).

## Discussion

### Interpretation of findings

We present a novel AI-based pipeline that automatically tracks bone lesions in PSMA-PET/CT scans. PET preselects follow-up lesions, simplifying CT-based localization - especially useful for densely clustered lesions. The method mirrors a physician’s comparison of baseline and follow-up scans.

In our prove of concept study, PET and binary segmentation aided in lesion localization, but alone where not sufficient for reliable lesion tracking. CT patches performed better for lesion tracking as they provide relatively stable anatomical context, whereas PET signals are more variable during therapy and can mislead the Siamese CNN. Although only CT patches are used for the Siamese network, the method remains PET/CT-based, as lesion selection and patch positioning rely on PET data—unlike CT-only approaches.

Contrary to expectations, a 2D Siamese CNN outperformed its 3D counterpart in lesion tracking. This can be attributed to the case distinction algorithm, which already encodes 3D spatial relationships during patch extraction. While 3D patches offer additional contextual data, it overlaps with existing inputs and adds complexity without improving performance. Furthermore, smaller 3D patches (50$\times$50$\times$5) yielded better results than larger ones (50$\times$50$\times$11), suggesting excess information can be detrimental. Overall, using 2D patches with a 2D network is a more efficient and effective solution for the presented PET/CT lesion tracking approach.

While the final network achieves a high overall accuracy, with particularly strong performance in tracking corresponding lesions - including substantial morphological and uptake changes - it still shows only limited performance in detecting new lesions. Improving this aspect is essential for future work, as the identification of new lesions plays a critical role in therapy response assessment [4].

### Comparison to existing lesion tracking methods

In our AI-based PET/CT lesion tracking approach, the network achieved an accuracy of 83.3%, comparable to existing CNN-based CT methods, despite the challenge of densely clustered bone lesions. Hering et al. [16] reported 80% accuracy for whole-body soft-tissue melanoma lesions, while Rafael-Palou et al. [21] achieved 88.8% re-identification of pulmonary nodules using a Siamese network. Among corresponding lesions, our method achieved 89.3%. Given the morphological variability and high number of palliative prostate cancer lesions under [^177^Lu]Lu-PSMA therapy - often greater than in melanoma or pulmonary cases - this represents a significant advancement, particularly as Rafael-Palou et al. also used a Siamese architecture [21].

Recent neural network methods like SimU-Net [25] report higher accuracies - 99.7% for lung and 95% for liver datasets. However, this approach is limited to lesions visible on CT and does not address whole-body lesion distribution. It also requires additional algorithms to handle complex changes such as fused or split lesions. Split lesions, in particular, challenge integrated follow-up segmentation methods [16]. Our method overcomes this by passing lesion positions through a case distinction algorithm and independently classifying patch pairs with a Siamese network, enabling flexible one-to-many or null assignments for robust tracking of complex developments.

A direct comparison to Yip et al.’s articulated registration method [8] is difficult, as they report only lesion overlap, not tracking accuracy. Santoro-Fernandes et al. achieved higher accuracies - 98% [9] and 90% [10] - but on datasets with far fewer lesions per scan. Assuming that one corresponding lesion matching decision counts for each one lesion in the baseline and follow-up scan, their FDG/FLT dataset averages 7.4 lesions per scan [9] - over seven times fewer than our prostate cancer dataset, which averages 57.6 lesions per scan. In their neuroendocrine tumor extension (41.6 lesions/scan), tracking accuracy dropped to 90% overall and 84% for corresponding lesions [10]. Our pipeline achieves 89% for corresponding lesions, surpassing these results despite the higher lesion burden. The 90% accuracy in their registration-based approach is largely due to 96% accuracy in detecting new or disappeared lesions [10]. In contrast, our AI method currently identifies new lesions at only 38% accuracy, which remains the main limitation affecting overall performance.

### Strengths and limitations for clinical use

The strength of the proposed pipeline lies in its ability to achieve reliable tracking accuracy on a dataset with an unprecedented density of lesions per patient, achieving high-performing tracking even for corresponding lesions also exhibiting substantial morphological and uptake changes. The robustness of the approach is further supported by its successful training and testing on a heterogeneous dataset - including images from various scanners and with differing slice thicknesses (Section Dataset) - highlighting its potential for use in real-world clinical settings. However, the primary limitation of the current pipeline is its low performance in detecting new lesions, which presently hinders its readiness for direct clinical application.

The lower accuracy in detecting new lesions in our AI-based pipeline stems from both the dataset and the tracking approach. The dataset includes heavily metastasized patients with many small, closely spaced new or disappeared lesions - often only a few voxels near existing lesion pairs. Even human experts struggle with closely located lesions, making the gold standard imperfect. As shown in Fig. 7C, the CNN actually outperformed a nuclear physician concerning a new lesion detection. For the clear cases of isolated new lesions, the Siamese CNN worked successfully. Creating a reliable reference dataset is inherently difficult due to inter-reader variability - especially for many small, adjacent lesions [40]. Since our dataset was annotated by a single nuclear medicine physician, this represents a study limitation.

A limitation stemming from the analysis pipeline is the simple thresholding approach used by the qPSMA software [26] for lesion delineation which can results in image noise being segmented as lesions or nearby lesions being fused into a large coalescent lesion. Future work will optimize segmentation for the pipeline by applying AI methods and incorporate lesion-organ localization into tracking.

One possible approach offering higher specificity for new lesions is the clustering method by Santoro-Fernandes et al. [9, 10]. A combined metric including this method for detection of new lesions and the Siamese CNN for identifying corresponding lesions may further improve performance. Future improvements may also include transformer networks to enhance anatomical precision [23].

In summary, the proposed PET/CT lesion tracking pipeline achieves an overall accuracy of 83%, with 89% accuracy for corresponding lesions and 38% for new lesions. Even without the discussed improvements, the achieved efficient identification of corresponding lesions over time enables new approaches for response assessment. Changes in PSMA radioligand uptake are a strong prognostic factor in patients with metastatic prostate cancer. However, these changes are currently only measured for a limited number of index lesions identified by a human observer. In the typical advanced prostate cancer patient with multiple osseous and lymph node metastases, these measurements encompass only a very small fraction of the metastases. Our AI based approach enables clinical studies that measure of changes in radioligand uptake after therapy for many more lesions. In contrast, to extensively studied approaches that look at changes in the average ligand uptake of multiple lesions, our approach allows researchers to assess the heterogeneity of response (i.e. radioligand uptake of some lesions decreasing and of others increasing). The pipeline may also help distinguish tumor development across different organs. This additional information may improve the prognostic value of PSMA PET scans for response assessment.

However, due to the limited accuracy in detecting new lesions, full clinical deployment would still require a high level of human supervision. The model’s brittleness primarily arises in cases with very small new lesions and high lesion density, where adjacent lesions are difficult to separate, even for expert readers. Given no clearly defined benchmark for clinical lesion tracking, the study by Huff et al. [40] offers a reference point: it found no significant difference between manual tracking by two nuclear medicine physicians and the automated method by Santoro-Fernandes et al. [9], which reported 90% accuracy. This may serve as a practical benchmark, indicating that our performance in tracking corresponding lesions approaches physician-level accuracy, while new lesion detection remains the main limitation. Following this proof of concept, and with future improvements in detecting new lesions, the pipeline could be integrated as a decision-support tool in clinical workflows. The developed principle of AI lesion tracking is not specific to bone lesions in PSMA-PET/CT scans and can be extended to other types of PET/CT studies. While the current dataset already includes variability from two tracers and scanner types, future studies will focus on validating the pipeline using multi-center data, which poses additional challenges due to greater variation in acquisition protocols, image quality, and annotation standards across institutions.

## Conclusion

In this paper, we address the gap in AI lesion tracking for PET/CT scans by employing a pipeline that includes segmentation, registration, a case distinction algorithm, and a Siamese Convolutional Neural Network. We provides first proof-of-concept data for an AI approach efficiently that can track the numerous lesions detected by PSMA-PET/CT technology in patients with advanced prostate cancer. The fully automated nature without manual lesion selection handling a large number of lesions per patient represents an added value to current registration-based PET/CT or AI-based CT lesion tracking tools. However, the performance for detecting new or disappeared lesions still needs to be improved. The developed method does not rely on characteristics of PSMA-PET/CT and may be applied to any oncological PET/CT.

## Supplementary Information

Below is the link to the electronic supplementary material.

Supplementary file 1 (pdf 199 KB)

## Data Availability

The datasets generated and analyzed during the current study are not publicly available due to privacy rights and data protection regulations.
