# Supplementary material for: AI lesion tracking in PET/CT imaging: a proposal for a Siamese-based CNN pipeline applied to PSMA PET/CT scans
Source: Eur J Nucl Med Mol Imaging. 2025 Jul 8;53(1):429–41. doi: 10.1007/s00259-025-07426-5 (PMC12660441; doi:10.1007/s00259-025-07426-5)
Supplement: Supplementary file 1 — (pdf 199 KB) [file 259_2025_7426_MOESM1_ESM.pdf]

Supplements

Article title: Towards AI Lesion Tracking in PET/CT Imaging: A Siamese-based CNN Pipeline applied on PSMA PET/CT Scans

Journal: European Journal of Nuclear Medicine and Molecular Imaging

Authors: Stefan P. Hein*, Manuel Schultheiss, Andrei Gafita, Raphael Zaum, Farid Yagubbayli, Robert Tauber, Isabel Rauscher, Matthias Eiber, Franz Pfeiffer, Wolfgang A. Weber

***Corresponding author:** stefan.hein@tum.de; Department of Nuclear Medicine, Technical University of Munich, Munich, 81675, Germany.

Image Registration

Since the body shape of the patients possibly changes in the course of therapy, an affine registration is performed in the pipeline that also allows for shearing and scaling in addition to the rotation and translation of a rigid transformation. The follow-up CT scan is registered towards the baseline CT scan. As a result we obtain a shear and rotation matrix $\mathbf{A} = (a_{11}, ...\ a_{33})$ and the translation vector $\mathbf{t} = (t_1, t_2, t_3)$, along with the center of rotation $\mathbf{c} = (c_1, c_2, c_3)$, defined as the center of the baseline scan. The transformation is then applied on the follow-up PET scan as well as on its 3D bone segmentation mask.

For the relocalization during patch extraction, positions $\mathbf{x}$ in the baseline and follow-up original scans can be projected into the respective other scan using the forward transformation $\mathbf{T}(\mathbf{x})$ or the reverse transformation $\mathbf{T^{-1}}(\mathbf{x})$ by applying the inverse shear and rotation matrix $\mathbf{A}^{-1}$.

$$\mathbf{T}:\ \mathbf{x}_{follow-up} = \mathbf{A} \cdot (\mathbf{x}_{\ baseline} - \mathbf{c}) + \mathbf{t} + \mathbf{c} \tag{1}$$

$$\mathbf{T^{-1}}:\ \mathbf{x}_{\ baseline} = \mathbf{A}^{-1} \cdot (\mathbf{x}_{follow-up} - \mathbf{c} - \mathbf{t}) + \mathbf{c} \tag{2}$$

For image registration SimpleElastix [1] was used.

Patch Extraction

The patches in this study were obtained by cropping axial layers from a whole-body dataset. For a 2D patch around a point of interest $p(x, y, z)$, the z-position selects the axial layer of the dataset, which consists of a CT scan, PET scan, and binary lesion segmentation. The scans are cropped around the x-y-position within a 50×50 pixel frame, as shown in figure 2. In 3D, several axial slices are included in the patch. For the experiments, 3D patch sizes of 50×50×5 pixels and 50×50×11 pixels were chosen. The extraction position is determined in the same way as for 2D patches, with the only difference being that for 50×50×5 pixel patches, two axial layers above

and below the determined z-position are added to the patch, and for 50×50×11 pixel patches, respectively, five axial layers above and below. As described in section 3.1, the resolution in x- and y-direction is higher than in z-direction and thus a pixel in an x-y-plane displays a smaller area than those in z-direction.

When creating a two-dimensional or a small three-dimensional patch out of a three-dimensional lesion, the position of the extraction is of high importance. Extracting the patch only at the Center of Mass (CoM) of each lesion and then creating patch pairs can lead to unsuitable true patch pairs that do not show the same anatomical region, even though they represent corresponding lesions. This is the case when a baseline lesion divides into several small follow-up lesions, as shown in figure 3A. When comparing a relatively small to a large lesion, the two centers of mass are on different axial layers, even though they are corresponding lesions. Therefore, the ROI of the larger lesion, within the comparison with the smaller one, is not its CoM. Instead, its ROI is represented by the anatomical environment of the smaller lesion. It is, thus, necessary to transfer the z-coordinate of the second CoM into the large lesion and extract a patch around the larger lesion at the adjusted axial layer. Hence, the lesion patches cannot be extracted independently. It is always necessary to extract a patch pair together, as the patch position for a lesion depends both on its own structure and on that of the compared lesion.

An algorithm uses hierarchical cases applied sequentially to determine the patch extraction point (fig. 3B). The 3D CoM of a lesion is used as a starting point and centered within the 2D axial slice of the lesion.

Case I projects the corrected CoM of the follow-up lesion into the baseline lesion using $\mathbf{T^{-1}}(\mathbf{x})$ (eq. 2), mostly used if the baseline lesion is larger and has either shrunk in volume or has divided into several smaller follow-up lesions.

Case II is implemented if Case I is not successful, projecting the corrected CoM of the baseline lesion into the follow-up lesion ($\mathbf{T}(\mathbf{x})$) (eq. 1), mostly applied with the baseline lesion being the smaller one.

Case III is utilized if due to shifts between the baseline and follow-up lesions the transferred CoMs cannot be identified within the lesions, mostly due to minor registration errors. To address this challenge, the algorithm locates the intersection of both lesions and designates the center of the overlap as the patch point for each lesion. The overlap is then transformed using $\mathbf{T}(\mathbf{x})$ (eq. 1) to determine its coordinates in the follow-up scan.

Even though *Cases I* to *III* mostly extract true lesion pairs, sometimes, due to registration errors, they may yield false patch pairs. Therefore, the use of the Siamese CNN approach remains relevant.

Case IV is designed for lesions that do not overlap, which can happen when corresponding lesions are not accurately registered. This method is also utilized for most false patch pairs. For the smaller lesion in the pair, the patch point is set at the axial layer of its CoM. For the larger lesion, the shape of the smaller lesion is projected onto the nearest end of the larger lesion in the z-direction. The point is then taken at

the layer where the CoM of the projected smaller lesion would be positioned. Mathematically, the z-coordinate of the extraction point of the larger lesion $z_{extr,l}$ can be expressed as follows:

$$z_{extr,l} = \begin{cases} z_{max,l} - \Delta z_s/2 & \text{if } z_{CoM,s} > z_{CoM,l} \\ z_{min,l} + \Delta z_s/2 & \text{if } z_{CoM,s} < z_{CoM,l} \ . \end{cases} \quad (3)$$

Here, Δz_s represents the height in the z-direction of the smaller lesion, and $z_{max,l}$ or $z_{min,l}$ indicates the z-coordinate of the highest or lowest point of the larger lesion. $z_{CoM,s}$ and $z_{CoM,l}$ denote the z-coordinates of the CoMs of the two lesions.

For the test set, a lesion from the baseline scan is compared to all follow-up lesions in a specific ROI of $\Delta x = 10$, $\Delta y = 10$ and $\Delta z = 5$ voxels around the margins of the projected baseline lesion ($\mathbf{T}(\mathbf{x})$, eq. 1). In z-direction the interval is smaller as its voxel size is usually larger than in x- and y- direction. This ROI was chosen as it contains 99% of the corresponding lesions, when tested on the train and validation set.

Reference Model

To validate the learning ability of the used network, the performance of the trained CNN is compared to that of a non-trainable model, which only compares the intensities of two 2D CT patches by subtracting them and taking the pixel-wise absolute value of the difference. This is equal to directly applying the $\mathbf{L_1}$-layer (main manuscript, eq. 1) without any CNN branches. The reference model is incapable of learning to compare anatomical structures. The decision output y of the intensity model with the input patches $\mathbf{X_1}$ and $\mathbf{X_2}$ of shape (N, N) can be described as:

$$y = 1 - \frac{1}{N^2} \sum_{i,j=1}^{N} \mathbf{L_1}_{i,j}(\mathbf{X_1}, \mathbf{X_2}) \quad (4)$$

Siamese Network

For the differently sized 3D patches with z=5 and z=11, the kernel size of the CNN had to be adapted as a larger patch can be further scaled down by larger kernels (supplemental table 1).

Applying the *Adam* optimizer [2], which already effectively adapts the learning rate due to momentum optimization, for our network case, we achieved best performance with an initial learning rate of 1×10^{-4}. Nevertheless, an additional combination of learning rate scheduling together with momentum optimization has shown good results [3]. In several siamese networks, different learning rate decay have been applied [4, 5]. For this reason, we chose *performance scheduling* for the learning rate with a patience of 5 epochs, a scheduling factor of 0.2 and a minimum learning rate of 1×10^{-5}.

As the lesion tracking task requires the comparison of different scans with possibly changed morphologies during therapy, a high grade of generalization is necessary. In order to achieve this and prevent overfitting, regularization is applied in various ways. In addition to the batch normalization, *ℓ^2-parameter regularization* is used

Table 1: Architecture Details of the used Siamese Branches

**Layer**	**Size 2D**	**Size 3D z5|z11**	**Kernels**	**Stride 2D (3D)**	**Output 2D**	**Output 3D z5|z11**
Input					50×50×1[a]	50×50×5|11×1[a]
Convolutional	5×5×1[b]	5×5×2|4×1[b]	20	1×1(×1)	46×46×20	46×46×4|8×20
Pooling	3×3	3×3×1|2	–	2×2(×1)	22×22×20	22×22×4|7×20
Convolutional	3×3×20	3×3×2|3×20	40	1×1(×1)	22×22×40	22×22×3|5×40
Pooling	2×2	2×2×1|2	–	2×2(×1)	11×11×40	11×11×3|5×40
Convolutional	3×3×40	3×3×2|3×40	50	1×1(×1)	9×9×50	9×9×2|3×50
Pooling	2×2	2×2×1|2	–	2×2(×1)	4×4×50	4×4×2|2×50

The table shows the structure of the layers for a 2D Siamese branch and for a 3D Siamese branch, which exists in two different versions for patches with z=5 and patches with z=11. Annotation example for the kernel size of the first 3D convolutional layer: 5×5×2×1 for patches with z=5 and 5×5×4×1 for patches with z=11.

[a] 1-channel patches as input. For 2-channel patches, the input shape is 50×50×2 (2D) and 50×50×5|11×2 (3D), respectively.

[b] First layer structure for 1-channel patches. For 2-channel patches, only the kernel size of first layer is extended to 5×5×2 (2D)and 5×5×4|2×2 (3D), respectively.

with a penalty factor of 5×10^{-4} as recommended for CNNs [6]. Following further suggestions for CNNs, a dropout rate of 40% is applied to the top three layers [7].

Supplemental table 2 displays the performance result of all combinations of the different network variations and the different patch types.

Table 2: Performance of the Siamese network

**Dim.**	**Patch Size**	**Patch Type**	**Validation Acc**	**Validation Loss**	**Test Accuracy**
2D	50×50	CT	0.790 ± 0.015	0.508 ± 0.030	0.830 ± 0.003
2D	50×50	CT/PET	0.800 ± 0.008	0.521 ± 0.016	0.806 ± 0.012
2D	50×50	CT/Seg	0.781 ± 0.010	0.530 ± 0.011	0.795 ± 0.001
2D	50×50	CT/Seg CT	0.795 ± 0.011	0.515 ± 0.013	0.788 ± 0.011
3D	50×50×5	CT	0.804 ± 0.008	0.486 ± 0.008	0.820 ± 0.008
3D	50×50×5	CT/PET	0.802 ± 0.002	0.499 ± 0.007	0.786 ± 0.019
3D	50×50×5	CT/Seg	0.819 ± 0.005	0.472 ± 0.010	0.790 ± 0.022
3D	50×50×5	CT/Seg CT	0.821 ± 0.011	0.475 ± 0.023	0.803 ± 0.012
3D	50×50×11	CT	0.829 ± 0.013	0.441 ± 0.016	0.786 ± 0.007
3D	50×50×11	CT/PET	0.817 ± 0.004	0.485 ± 0.006	0.761 ± 0.017
3D	50×50×11	CT/Seg	0.830 ± 0.004	0.463 ± 0.007	0.791 ± 0.017
3D	50×50×11	CT/Seg CT	0.836 ± 0.007	0.465 ± 0.016	0.790 ± 0.018

Seg: Binary Lesion Segmentation; Seg CT: Segmented CT

References

[1] Perera M, Papa N, Christidis D, Wetherell D, Hofman MS, Murphy DG, et al. Sensitivity, Specificity, and Predictors of Positive 68Ga–Prostate-specific Membrane Antigen Positron Emission Tomography in Advanced Prostate Cancer: A Systematic Review and Meta-analysis. Eur Urol. 2016;70:926–937. https://doi.org/10.1016/j.eururo.2016.06.021.

[2] Carlin BI, Andriole GL. The natural history, skeletal complications, and management of bone metastases in patients with prostate carcinoma. Cancer. 2000;88:2989–2994. https://doi.org/10.1002/1097-0142(20000615)88:12+⟨2989::aid-cncr14⟩3.0.co;2-q.

[3] Wahl RL, Jacene H, Kasamon Y, Lodge MA. From RECIST to PERCIST: Evolving considerations for PET response criteria in solid tumors. J Nucl Med. 2009;50:122–150. https://doi.org/10.2967/jnumed.108.057307.

[4] Dell'Oro M, Huff DT, Lokre O, Kendrick J, Munian Govindan R, Ong JS, et al. Assessing the Heterogeneity of Response of [68Ga] Ga-PSMA-11 PET/CT Lesions in Patients With Biochemical Recurrence of Prostate Cancer. Clin Genitourin Cancer. 2024;22:102155. https://doi.org/10.1016/j.clgc.2024.102155.

[5] Opfer R, Brenner W, Carlsen I, Renisch S, Sabczynski J, Wiemker R. Automatic lesion tracking for a PET/CT based computer aided cancer therapy monitoring system. In: Proc Medical Imaging 2008: Computer-Aided Diagnosis. SPIE; 2008;6915:691513. https://doi.org/10.1117/12.770356.

[6] Fox JJ, Autran-Blanc E, Morris MJ, Gavane S, Nehmeh S, Nuffel AV, et al. Practical Approach for Comparative Analysis of Multilesion Molecular Imaging Using a Semiautomated Program for PET/CT. J Nucl Med. 2011;52:1727–1732. https://doi.org/10.2967/JNUMED.111.089326.

[7] Yip S, Jeraj R. Use of articulated registration for response assessment of individual metastatic bone lesions. Phys Med Biol. 2014;59:1501–1514. https://doi.org/10.1088/0031-9155/59/6/1501.

[8] Santoro-Fernandes V, Huff D, Scarpelli ML, Perk TG, Albertini MR, Perlman S, et al. Development and validation of a longitudinal soft-tissue metastatic lesion matching algorithm. Phys Med Biol. 2021;66. https://doi.org/10.1088/1361-6560/ac1457.

[9] Santoro-Fernandes V, Huff DT, Rivetti L, Deatsch A, Schott B, Perlman SB, et al. An automated methodology for whole-body, multimodality tracking of individual cancer lesions. Phys Med Biol. 2024;69. https://doi.org/10.1088/1361-6560/ad31c6.

[10] Seifert R, Herrmann K, Kleesiek J, Schäfers M, Shah V, Xu Z, et al. Semiautomatically quantified tumor volume using 68Ga-PSMA-11 PET as a biomarker for survival in patients with advanced prostate cancer. J Nucl Med. 2020;61:1786–1792. https://doi.org/10.2967/jnumed.120.242057.

[11] Sibille L, Seifert R, Avramovic N, Vehren T, Spottiswoode B, Zuehlsdorff S, et al. (18)F-FDG PET/CT uptake classification in lymphoma and lung cancer by using deep convolutional neural networks. Radiology. 2020;294:445–452. https://doi.org/10.1148/radiol.2019191114.

[12] Schott B, Weisman AJ, Perk TG, Roth AR, Liu G, Jeraj R. Comparison of automated full-body bone metastases delineation methods and their corresponding prognostic power. Phys Med Biol. 2023;68. https://doi.org/10.1088/1361-6560/acaf22.

[13] Tan M, Li Z, Qiu Y, McMeekin SD, Thai TC, Ding K, et al. A new approach to evaluate drug treatment response of ovarian cancer patients based on deformable image registration. IEEE Trans Med Imag. 2016;35:316–325. https://doi.org/10.1109/TMI.2015.2473823.

[14] Dankerl P, Cavallaro A, Dietzel M, Tsymbal A, Kramer M, Seifert S, et al. Clinical evaluation of semi-automatic landmark-based lesion tracking software for CT-scans. Cancer Imaging. 2014;14:1–7. https://doi.org/10.1186/1470-7330-14-6.

[15] Hering A, Peisen F, Amaral T, Gatidis S, Eigentler T, Othman A, et al. Whole-body soft-tissue lesion tracking and segmentation in longitudinal CT imaging studies. In: Proc Mach Learn Res. ICLR; 2021;Vol. 143:312–326.

[16] Kuckertz S, Weiler F, Matusche B, Lukas C, Spies L, Klein J, et al. A system for fully automated monitoring of lesion evolution over time in multiple sclerosis. In: Proc SPIE Med Imag. SPIE; 2021;11597:553–558. https://doi.org/10.1117/12.2582156.

[17] Kuckertz S, Klein J, Engel C, Geisler B, Kraß S, Heldmann S. Fully automated longitudinal tracking and in-depth analysis of the entire tumor burden: unlocking the complexity. In: Proc SPIE Med Imag. SPIE; 2022;12033:455–459. https://doi.org/10.1117/12.2613080.

[18] Gomariz A, Li W, Ozkan E, Tanner C, Goksel O. Siamese networks with location prior for landmark tracking in liver ultrasound sequences. In: IEEE Int Symp Biomed Imaging. IEEE; 2019:1757–1760. https://doi.org/10.1109/ISBI.2019.8759382.

[19] Liu F, Liu D, Tian J, Xie X, Yang X, Wang K. Cascaded one-shot deformable convolutional neural networks: Developing a deep learning model for respiratory motion estimation in ultrasound sequences. Med Image Anal. 2020;65. https://doi.org/10.1016/j.media.2020.101793.

[20] Rafael-Palou X, Aubanell A, Bonavita I, Ceresa M, Piella G, Ribas V, et al. Re-identification and growth detection of pulmonary nodules without image registration using 3D siamese neural networks. Med Image Anal. 2021;67. https://doi.org/10.1016/j.media.2020.101823.

[21] Cai J, Tang Y, Yan K, Harrison AP, Xiao J, Lin G, et al. Deep lesion tracker: Monitoring lesions in 4D longitudinal imaging studies. In: Proc IEEE Comput Soc Conf Comput Vis Pattern Recognit. IEEE; 2021:15154–15164. https://doi.org/10.1109/CVPR46437.2021.01491.

[22] Tang W, Kang H, Zhang H, Yu P, Arnold CW, Zhang R. Transformer Lesion Tracker. In: Med Image Comput Comput Assist Interv – MICCAI 2022. Springer; 2022;LNCS Vol. 13436:196–206. https://doi.org/10.1007/978-3-031-16446-0_19.

[23] Szeskin A, Rochman S, Weiss S, Lederman R, Sosna J, Joskowicz L. Liver lesion changes analysis in longitudinal CECT scans by simultaneous deep learning voxel classification with SimU-Net. Med Image Anal. 2023;83:102675. https://doi.org/10.1016/j.media.2022.102675.

[24] Rochman S, Szeskin A, Lederman R, Sosna J, Joskowicz L. Graph-based automatic detection and classification of lesion changes in pairs of CT studies for oncology follow-up. International Journal of Computer Assisted Radiology and Surgery. 2024;19:241–251. https://doi.org/10.1007/s11548-023-03000-2.

[25] Gafita A, Bieth M, Krönke M, Tetteh G, Navarro F, Wang H, et al. qPSMA: Semiautomatic software for whole-body tumor burden assessment in prostate cancer using (68)Ga-PSMA11 PET/CT.. J. Nucl. Med.. 2019;60:1277–1283. https://doi.org/10.2967/jnumed.118.224055.

[26] Marstal K, Berendsen F, Staring M, Klein S. SimpleElastix: A user-friendly, multi-lingual library for medical image registration. In: Proc IEEE Comput Soc Conf Comput Vis Pattern Recognit. IEEE; 2016:574–582. https://doi.org/10.1109/CVPRW.2016.78.

[27] Koch G, Zemel R, Salakhutdinov R. Siamese neural networks for one-shot image recognition. In: ICML Deep Learning Workshop. ICML; 2015;Vol. 2.

[28] Kingma DP, Ba JL. Adam: A method for stochastic optimization. In: 3rd International Conference on Learning Representations. ICLR; 2015.

[29] Senior A, Heigold G, Yang K, Inc G. An empirical study of learning rates in deep neural networks for speech recognition. In: Proc IEEE Int Conf Acoust Speech Signal Process. IEEE; 2013:6724–6728.

[30] Zagoruyko S, Komodakis N. Learning to compare image patches via convolutional neural networks. In: Proc IEEE Comput Soc Conf Comput Vis Pattern Recognit. IEEE; 2015:4353–4361. https://doi.org/10.1109/CVPR.2015.7299064.

[31] Krizhevsky A, Sutskever I, Hinton GE. ImageNet classification with deep convolutional neural networks. Comm ACM. 2017;60:84–90. https://doi.org/10.1145/3065386.

[32] Szepesi P, Szilágyi L. Detection of pneumonia using convolutional neural networks and deep learning. Biocybern Biomed Eng. 2022;42:1012–1022. https://doi.org/10.1016/j.bbe.2022.08.001.

[33] Karimzadeh A, Heck M, Tauber R, Knorr K, Haller B, D'Alessandria C, et al. 177Lu-PSMA-I&T for treatment of metastatic castration-resistant prostate cancer: Prognostic value of scintigraphic and clinical biomarkers. J Nucl Med. 2023;64:402–409. https://doi.org/10.2967/jnumed.122.264402.

[34] Eder M, Schäfer M, Bauder-Wüst U, Hull WE, Wängler C, Mier W, et al. 68Ga-complex lipophilicity and the targeting property of a urea-based PSMA inhibitor for PET imaging. Bioconjug Chem. 2012;23:688–697. https://doi.org/10.1021/bc200279b.

[35] Wurzer A, Carlo DD, Schmidt A, Beck R, Eiber M, Schwaiger M, et al. Radiohybrid ligands: A novel tracer concept exemplified by 18F- or 68Ga-labeled rhPSMA inhibitors. J Nucl Med. 2020;61:735–742. https://doi.org/10.2967/jnumed.119.234922.

[36] Wurzer A, Parzinger M, Konrad M, Beck R, Günther T, Felber V, et al. Preclinical comparison of four [18F, natGa]rhPSMA-7 isomers: influence of the stereoconfiguration on pharmacokinetics. EJNMMI Research. 2020;10. https://doi.org/10.1186/s13550-020-00740-z.

[37] Eiber M, Maurer T, Souvatzoglou M, Beer AJ, Ruffani A, Haller B, et al. Evaluation of hybrid 68Ga-PSMA ligand PET/CT in 248 patients with biochemical recurrence after radical prostatectomy. J Nucl Med. 2015;56:668–674. https://doi.org/10.2967/jnumed.115.154153.

[38] Kroenke M, Schweiger L, Horn T, Haller B, Schwamborn K, Wurzer A, et al. Validation of 18F-rhPSMA-7 and 18F-rhPSMA-7.3 PET imaging results with histopathology from salvage surgery in patients with biochemical recurrence of prostate cancer. J Nucl Med. 2022;63:1809–1814. https://doi.org/10.2967/jnumed.121.263707.

[39] Gafita A, Rauscher I, Weber M, Hadaschik B, Wang H, Armstrong WR, et al. Novel Framework for Treatment Response Evaluation Using PSMA PET/CT in Patients with Metastatic Castration-Resistant Prostate Cancer (RECIP 1.0): An International Multicenter Study. J Nucl Med. 2022;63:1651–1658. https://doi.org/10.2967/jnumed.121.263072.

[40] Huff DT, Santoro-Fernandes V, Chen S, Chen M, Kashuk C, Weisman AJ, et al. Performance of an automated registration-based method for longitudinal lesion

matching and comparison to inter-reader variability. Phys Med Biol. 2023;68. https://doi.org/10.1088/1361-6560/acef8f.
